# A multi-center, open-label, two-part study to investigate the effect of renal function and hemodialysis on the pharmacokinetics of the novel β-lactamase inhibitor nacubactam

**DOI:** 10.1128/aac.00113-26

**Published:** 2026-04-30

**Authors:** Jun Morita, Kazuya Ishiwata, Shogo Matsumoto, Ignacio Rodriguez, Katie Patel, Gemma Attley, Navita L. Mallalieu, Darren Bentley

**Affiliations:** 1 Meiji Seika Pharma Co. Ltd., Tokyo, Japan; 2 Roche Pharma Research and Early Development, Roche Innovation Centre New York, Little Falls, New Jersey, USA; 3 Global Product Development, Roche Products Limited, Welwyn Garden City, United Kingdom; 4 Roche Pharma Research and Early Development, Roche Innovation Centre New York, New York, New York, USA; 5 Certara Drug Development Solutions, Certara, Sheffield, United Kingdom; Providence Portland Medical Center, Portland, Oregon, USA

**Keywords:** β-lactamase inhibitor, ESRD, nacubactam, pharmacokinetics, renal impairment

## Abstract

Nacubactam is a novel bicyclic non-β-lactam β-lactamase inhibitor that acts as a penicillin-binding protein (PBP-2) active antibacterial, almost exclusively cleared by renal excretion. This study assessed the effect of renal function and hemodialysis on nacubactam pharmacokinetics following a single intravenous (IV) 1 g infusion. Part 1 included three subjects with normal renal function, and six each with mild, moderate, or severe renal impairment; Part 2 included eight subjects with end-stage renal disease undergoing hemodialysis. We observed a strong linear relationship between renal function and both nacubactam total clearance and renal clearance. Compared with subjects with normal renal function, mean nacubactam total clearance was 49%, 73%, and 85% lower among subjects with mild, moderate, and severe renal impairment, respectively, and renal clearance was 42%, 70%, and 90% lower. Nacubactam systemic exposure was correspondingly greater in subjects with impaired renal function. Compared with the normal renal function group, AUC_0-inf_ was 2-fold, 3.7-fold, and 6.6-fold higher in the mild, moderate, and severe impairment groups, respectively. Similar results were observed across four different methods of estimating renal clearance. Nacubactam was cleared by hemodialysis, with more than 60% of a single dose eliminated during a 4-h dialysis session. Renal impairment had no apparent effect on the safety profile of nacubactam, and a single 1 g dose was well tolerated in all groups. These data can be used to underwrite nacubactam dosing recommendations for patients with impaired renal function, including those receiving renal replacement therapy.

**CLINICAL TRIALS:**

This study is registered with ClinicalTrials.gov as NCT02975388.

## INTRODUCTION

During the past century, the discovery and clinical administration of β-lactam antibiotics revolutionized the treatment of infection (1) although their effectiveness against Gram-negative bacterial infections subsequently diminished with the rapid evolution of antimicrobial drug resistance (AMR), giving rise to a major global health concern (2). More recently, combining β-lactams with β-lactamase inhibitors (BLIs) has proven to be a successful strategy to protect against the enzymatic modifications which result in β-lactam inactivation, thereby restoring antibiotic effectiveness (3). There is now considerable evidence to support the efficacy and safety of various β-lactam/BLI combinations during the treatment of Gram-negative bacterial infections such as complicated urinary tract infections (cUTI), complicated intra-abdominal infections (cIAI), and hospital-acquired and ventilator-associated pneumonia (VABP/HABP) (4). However, given that the main elimination pathway for both β-lactams and BLIs is via renal excretion, dose adjustment is often necessary for patients with renal impairment to ensure adequate antibiotic exposure, although exceptions may exist depending on the severity of impairment, and also to prevent renal toxicity and acute kidney injury (5, 6).

Nacubactam (OP0595, RO7079901, and former RG-6080) is a novel bicyclic non-β-lactam BLI being developed to treat serious Gram-negative bacterial infections. The dual mechanism of action of nacubactam (inhibition of serine β-lactamases classes A, C, and D, plus inhibition of penicillin-binding protein 2 in *Enterobacteriaceae*) means that it has antibacterial activity and also protects and enhances the activity of partner β-lactam antibiotics (7–9). A Phase 3 study of nacubactam in patients with cUTI or acute uncomplicated pyelonephritis (Integral-1; NCT04675996) recently completed and met the primary endpoint (10); another Phase 3 study in patients with carbapenem-resistant *Enterobacteriaceae* infections (Integral-2 study; NCT05905055) is ongoing.

Previous pharmacokinetic (PK) studies demonstrated that nacubactam is almost exclusively cleared by renal excretion (11), and it was anticipated that renal function would have a major influence on nacubactam PK. Therefore, the principal aim of this study was to quantify the effect of reduced renal function and hemodialysis on the PK of a single IV dose of nacubactam.

## RESULTS

### Study population

A total of 21 subjects were enrolled in Part 1, three with normal renal function, and six each with mild, moderate, or severe renal impairment. A further eight subjects with end-stage renal disease (ESRD) undergoing hemodialysis were enrolled in Part 2. All subjects received study drug (nacubactam) and were included in the safety and PK analysis populations (Fig. S1). Although the study protocol specified that a maximum of six subjects with normal renal function could be enrolled, only three were actually dosed with nacubactam. This was due to the observation, based on interim data from subjects with renal impairment, that there was a clear relationship between nacubactam clearance and renal function. Consequently, it was decided to limit the number of subjects with normal renal function, as it was considered that exposure of further healthy individuals to nacubactam would not meaningfully alter the outcome of the key PK analyses.

Baseline demographic and clinical characteristics are reported in Table 1. Subjects in Part 1 were predominantly female (14/21; 66.7%) and white (18/21; 85.7%), with ages ranging from 25 to 79 years. Those in Part 2 were predominantly male (5/8; 62.5%) and Black or African-American (5/8; 62.5%) and aged between 28 and 63 years.

**TABLE 1:** Baseline characteristics of study participants who received a single 1 g IV dose of nacubactam^*a*^

	Group according to renal function/impairment				
	Normal (*N* = 3)	Mild (*N* = 6)	Moderate (*N* = 6)	Severe (*N* = 6)	ESRD (*N* = 8)
Age, years					
Mean (SD)	33.3 (8.5)	66.2 (7.9)	67.2 (10.7)	72.2 (4.0)	51.0 (11.5)
Median (range)	33.0 (25–42)	65.0 (57–79)	68.5 (53–79)	73.0 (66–76)	51.0 (28–63)
Sex, *n* (%)					
Male	1 (33.3)	3 (50.0)	1 (16.7)	2 (33.3)	5 (62.5)
Female	2 (66.7)	3 (50.0)	5 (83.3)	4 (66.7)	3 (37.5)
Race, *n* (%)					
White	2 (66.7)	5 (83.3)	5 (83.3)	6 (100)	2 (25.0)
African-American	1 (33.3)	1 (16.7)	1 (16.7)	0	5 (62.5)
Other	0	0	0	0	1 (12.5)
Ethnicity, *n* (%)					
Hispanic or Latino	0	2 (33.3%)	1 (16.7%)	2 (33.3%)	0
Not Hispanic or Latino	3 (100%)	4 (66.7%)	5 (83.3%)	4 (66.7%)	8 (100%)
Body weight, kg					
Mean (SD)	72.60 (8.27)	70.20 (9.83)	73.82 (6.05)	76.45 (17.04)	89.76 (26.18)
BMI, kg/m^2^					
Mean (SD)	25.9 (1.0)	26.4 (2.6)	27.8 (2.5)	28.4 (5.9)	30.3 (6.7)
Temperature, °C					
Mean (SD)	36.8 (0.1)	36.1 (0.4)	36.6 (0.5)	36.6 (0.4)	36.5 (0.6)

^*a*^ ESRD, end-stage renal disease; SD, standard deviation.

### Part 1: effect of renal impairment on nacubactam PK

**Fig 1:**
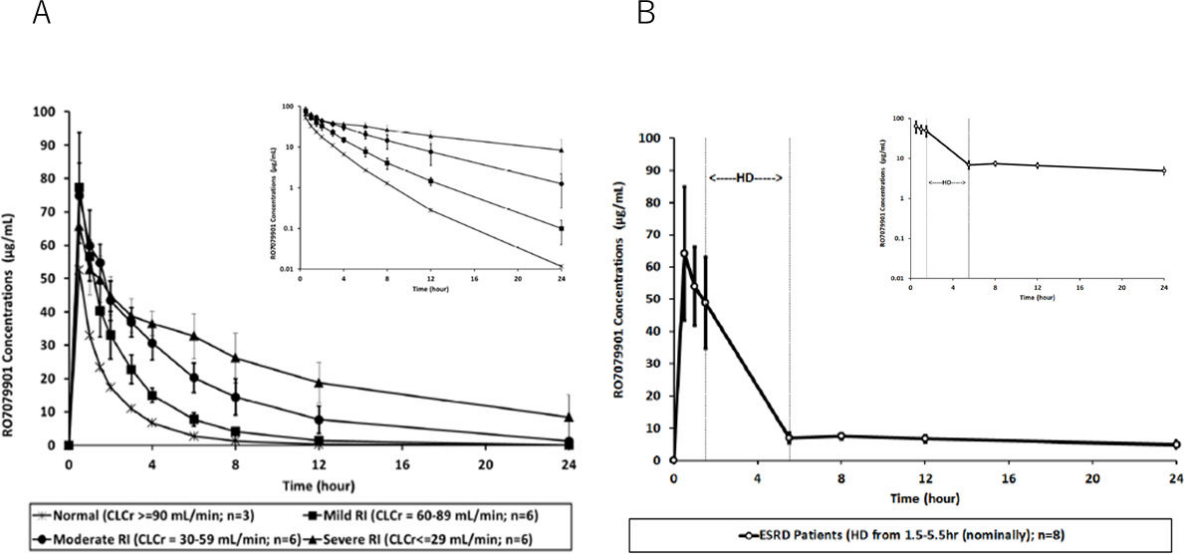
Mean (±SD) plasma concentration-time profiles of nacubactam in Part 1 (**A**). Data are shown on a linear scale with an inset panel showing the log scale. Part 2 (**B**) of the study. Data are shown on a linear scale with an inset panel showing the log scale.

A summary of derived nacubactam PK parameters by renal function group is presented in Table 2. Mean plasma concentration vs time profiles for nacubactam are presented by renal function group in Fig. 1.

**TABLE 2:** Summary of selected pharmacokinetic parameters following a single 1 g IV dose of nacubactam during Part 1^*a*^^,^^*b*^

	Group according to renal function/impairment			
	Normal (*N* = 3)	Mild (*N* = 6)	Moderate (*N* = 6)	Severe (*N* = 6)
CL, L/h	10.0 (10)	5.1 (12)	2.8 (23)	1.5 (37)
CL_r_, L/h	8.0 (24)	4.6 (22)	2.2 (22)	0.8 (78)
*V*_ss_, L	21.4 (9)	15.2 (21)	14.9 (11)	18.3 (13)
AUC_0–8_, µg.h/mL	95 (10)	175 (14)	263 (13)	302 (10)
AUC_0–24_, µg.h/mL	100 (10)	196 (12)	356 (22)	546 (20)
AUC_0–inf_, µg.h/mL	100 (10)	196 (12)	363 (23)	661 (37)
*C*_max_, µg/mL	52.2 (14)	77.5 (19)	74.3 (15)	65.2 (18)
*T*_max_, h	0.5 (0.5–0.5)	0.5 (0.5–1.0)	0.5 (0.5–0.5)	0.5 (0.5–1.0)
*t*_1/2_, h	2.41 (7)	2.89 (8)	4.32 (16)	9.00 (37)
Ae_0–24_, mg	791 (19)	899 (23)	742 (10)	417 (55)
Fe%_0–24_, %	79.1 (19)	89.9 (23)	74.2 (10)	41.7 (55)

^*a*^ Data are shown as geometric mean (coefficient of variation %), except *T*_max_ which is shown as median (range).

^*b*^ Ae_0–24_, cumulative amount excreted in urine from time 0 to 24 h; AUC_0–8_, area under the plasma concentration-time curve from time 0 to 8 h; AUC_0–24_, area under the plasma concentration-time curve from time 0 to 24 h; AUC_0–inf_, area under the concentration–time curve from time 0 to infinity; CL, total clearance (based on dose/AUC_0–inf_); CL_r_, renal clearance (based on Ae_0–24_/AUC_0–24_); *C*_max_, maximum observed plasma concentration; Fe%_0–24_, cumulative percentage excreted in urine from time 0 to 24 h; *T*_max_, time to maximum concentration; *t*_1/2_, terminal half-life; *V*_ss_, volume of distribution at steady-state.

**Fig 2:**
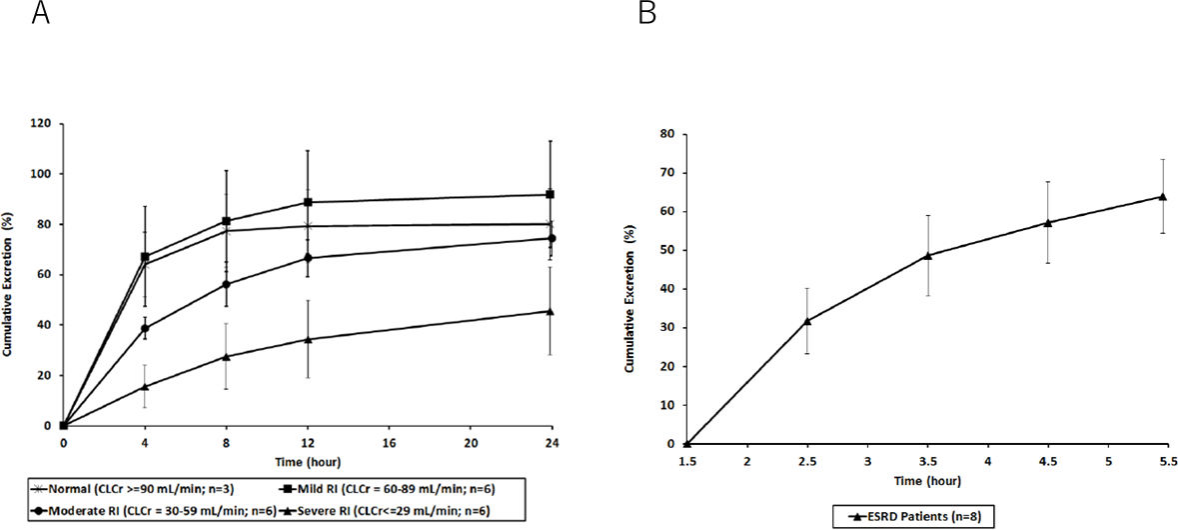
Mean (±SD) cumulative excretion (from time 0 to *t* h) of nacubactam in Part 1 (**A**) of the study. Data are shown as a percentage of the administered dose. Part 2 (**B**) of the study. Data are shown as a percentage of the administered dose. ESRD, end-stage renal disease; HD, hemodialysis; SD, standard deviation.

Mean cumulative excretion profiles in urine (as a percentage of dose) are presented in Fig. 2. In one subject with moderate renal impairment, the amount of drug excreted unchanged was 173% of the administered dose. There was no evidence of incorrect dosing, incorrect recording of urine collection volumes, or analytical errors. As excretion of more than 100% of the administered dose is physiologically implausible, the urinary excretion data and associated PK parameters for this subject were excluded from the summary analyses. Data from the remaining subjects indicated that nacubactam clearance from plasma was slower according to the degree of renal impairment, but the majority had been eliminated by 24 h post-dose. Urinary excretion was correspondingly affected by renal impairment, with a markedly lower percentage of administered nacubactam excreted by 24 h post-dose in the severe group compared with the other groups.

### Part 2: effect of hemodialysis on nacubactam PK

Nacubactam PK parameters in subjects with ESRD are presented in Table 3. Two subjects had extremely high maximal plasma concentrations at the end of infusion. The samples were confirmed to have been taken from the same arm as that used to infuse the study drug; therefore, these samples and the associated PK parameters were excluded from the summary results. Mean nacubactam plasma concentration vs time profiles in subjects with ESRD are presented in Fig. 1.

**TABLE 3:** Summary of selected pharmacokinetic parameters following a single 1 g dose of nacubactam during Part 2^*a*^^,^^*b*^

	Subjects with ESRD (*N* = 8)
CL_D(REC)_, L/h	9.56 (21)
CL_D(ER)_, L/h	11.3 (11)
AUC_HD_, µg.h/mL	66.2 (10)
AUC_0–inf_, µg.h/mL	443 (16)
*C*_max_, µg/mL	63.4 (30)
*T*_max_, h	0.5 (0.5–1.0)
*t*_1/2_, h	20.6 (36)
Ae_HD_, mg	632 (16)
Fe%_HD_, %	63.2 (16)

^*a*^ Data are shown as geometric mean (coefficient of variation %), except *T*_max_ which is shown as median (range).

^*b*^ Ae_HD_, cumulative amount excreted in dialysis fluid during hemodialysis period; AUC_0–inf_, area under the concentration–time curve from time 0 to infinity; AUC_HD_, area under the plasma concentration-time curve during the dialysis period; CL_D(ER)_, dialysis clearance estimated from extraction ratio across the dialyzer; CL_D(REC)_, dialysis clearance estimated from recovery in dialysis fluid; *C*_max_, maximum observed plasma concentration; ESRD, end-stage renal disease; Fe%_HD_, cumulative percentage excreted in dialysis fluid during hemodialysis period; *T*_max_, time to maximum concentration; *t*_1/2_, terminal half-life.

Mean cumulative excretion profiles in dialysis fluid (as a percentage of dose) are presented in Fig. 2. Three subjects with ESRD provided only a single urine sample; thus, no urinary PK parameters could be determined for these subjects. In the remaining subjects with ESRD, nacubactam was appreciably cleared during hemodialysis (median duration, 4 h; range, 2.8–4.0 h). By the end of the hemodialysis session (i.e., ~5.5 h [median] post-infusion start), the plasma concentration of nacubactam had fallen to a mean of around 7 µg/mL (coefficient of variation ~22%). On average, 63% (range: 49%–74%) of the administered dose of nacubactam was excreted into the dialysate during the hemodialysis session. Hemodialysis clearance based on recovery of nacubactam in the dialysis fluid was 9.6 L/h (range: 6.9–11.5 L/h). The geometric mean dialysis clearance estimated from extraction ratio across the dialyzer was 11.3 L/h (range: 8.8–12.6 L/h).

### Relationships between nacubactam PK and renal function

**Fig 3:**
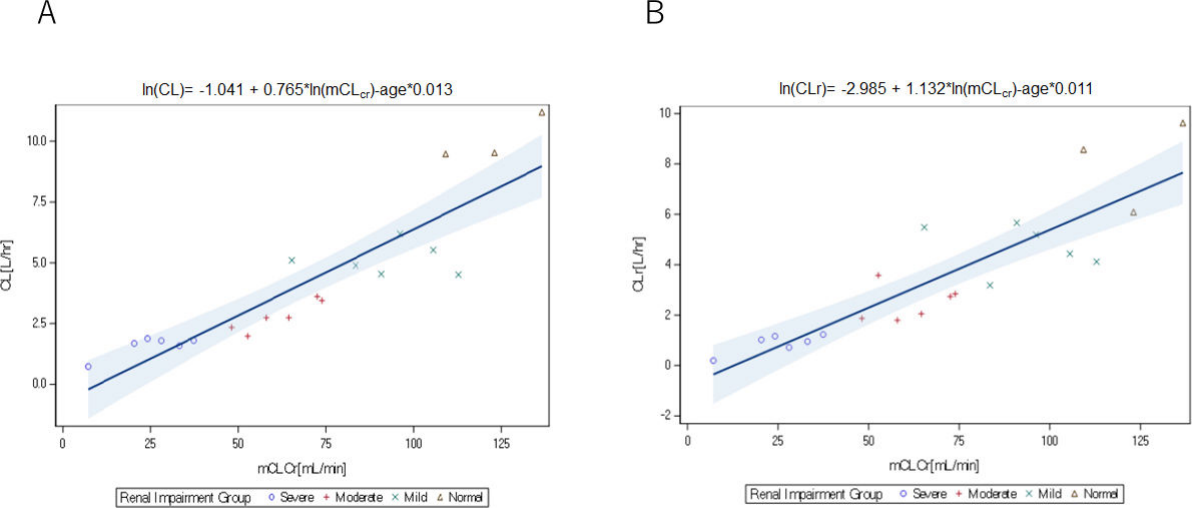
Scatterplot of nacubactam CL (**A**) and CL_r_ (**B**) against mCL_Cr_ (linear scale). CL, total clearance; CL_r_, renal clearance; ln, natural log; mCL_Cr_, measured creatinine clearance.

The relationships between the nacubactam PK parameters of total clearance (CL) and renal clearance (CL_r_) and renal function (using various measures) were explored in Part 1 (Table 4). Scatterplots showing estimates of nacubactam CL and CL_r_ for each individual plotted against corresponding measured creatinine clearance (CL_Cr_) values are presented in Fig. 3. Strong positive relationships were observed in each case, and the estimated slopes of the lines describing these relationships were statistically significantly different from zero (*P* < 0.0001). The observed data points were tightly distributed around the predicted mean for CL and CL_r_, and for both parameters, the majority of variability was captured by the independent variable (*r*^2^ = 90.8% and 91.4%, respectively). Neither body weight nor sex was identified as statistically significant covariates for relationships between CL or CL_r_ and measured CL_Cr_. Age was identified as a statistically significant covariate although the effect size was modest (<1.3% reduction in nacubactam CL and CL_r_ for each additional year of age).

**TABLE 4:** Summary of linear regression analyses parameter estimates (Part 1)^*a*^^,^^*b*^

Independent variable	Intercept (*µ*)	Slope (*β*)	*r* ^2^	Covariates
Dependent variable: nacubactam CL, L/h				
mCL_Cr_	−1.041	0.765	0.908	Age (−0.013)
eCL_Cr(CG)_	−2.603	0.989	0.928	–
eCL_Cr(Jeliffe)_	−3.620	1.008	0.929	Weight (0.015); female (−0.221)
eGFR_(MDRD)_	−0.610	0.709	0.898	Age (−0.013)
eGFR_(CKD-EPI)_	−3.020	0.954	0.981	Weight (0.010)
Dependent variable: nacubactam CL_r_, L/h				
mCL_Cr_	−2.985	1.132	0.914	Age (−0.011)
eCL_Cr(CG)_	−4.116	1.305	0.882	–
eCL_Cr(Jeliffe)_	−6.307	1.372	0.926	Weight (0.027)
eGFR_(MDRD)_	−6.511	1.292	0.910	Weight (0.031); female (0.455)
eGFR_(CKD-EPI)_	−3.251	1.117	0.846	–

^*a*^ Regression analyses were performed on the natural log scale.

^*b*^ CG, Cockcroft-Gault; CKD-EPI, Chronic Kidney Disease-Epidemiology; CL, total clearance; CL_r_, renal clearance; eCL_Cr_, estimated creatinine clearance; eGFR, estimated glomerular filtration rate; mCL_Cr_, measured creatinine clearance; MDRD, Modification of Diet in Renal Disease; –, each variable is not significant.

There were strong positive relationships between nacubactam clearance and renal function for all assessment methods used (Table 4), and in each case, the majority of variability was captured by the independent variables (i.e., *r*^2^ ≥ 85%).

Scatterplots showing estimates of individual nacubactam volume of distribution (*V*_d_) at steady state against measured and estimated CL_Cr_ are shown in Fig. S2; no apparent relationships were observed.

An analysis of variance (ANOVA) model fitted to log transformed parameters was used to estimate differences in nacubactam PK parameters (CL, CL_r_, and AUC_0–inf_) between renal function groups (Table 5; Fig. S3). Compared with subjects with normal renal function, mean nacubactam CL was 49%, 73%, and 85% lower, and CL_r_ was 42%, 70%, and 90% lower, among subjects with mild, moderate, and severe renal impairment, respectively. AUC_0–inf_ was 2-fold, 3.7-fold, and 6.6-fold higher among subjects with mild, moderate, and severe renal impairment, respectively, than in subjects with normal renal function.

**TABLE 5:** Summary of ANOVA comparisons of nacubactam PK parameters between renal function groups^*a*^^,^^*b*^

Independent variable	Normal vs mild	Normal vs moderate	Normal vs severe
CL	0.508 (0.377, 0.684)	0.274 (0.204, 0.369)	0.151 (0.112, 0.203)
CL_r_	0.578 (0.342, 0.977)	0.302 (0.179, 0.511)	0.096 (0.057, 0.163)
AUC_0–inf_	1.97 (1.46, 2.65)	3.65 (2.71, 4.91)	6.64 (4.93, 8.94)

^*a*^ Data are shown as geometric mean ratios (95% confidence intervals).

^*b*^ ANOVA, analysis of variance; AUC_0–inf_, area under the concentration–time curve from time 0 to infinity; CL, total clearance; CL_r_, renal clearance; PK, pharmacokinetics.

### Safety

Overall, a single IV infusion of nacubactam was well tolerated, and no significant safety findings were documented in any of the subjects dosed. Adverse events (AEs) are summarized in Table 6. In Part 1, 4/21 (19.0%) subjects experienced AEs, and in Part 2, 1/8 (12.5%) had an AE. None of the AEs were considered related to study treatment, and all recovered without sequelae.

**TABLE 6:** Summary of AEs^*a*^

	Group according to renal function/impairment				
	Normal (*N* = 3)	Mild (*N* = 6)	Moderate (*N* = 6)	Severe (*N* = 6)	ESRD (*N* = 8)
Subjects with any AE	0	1	2	1	1
Subjects with severe AE	0	0	0	1	0
Subjects with serious AE	0	0	0	1	0
Deaths	0	0	0	0	0
AE (preferred term), number of events					
Atrial fibrillation	0	0	0	1	0
Conjunctival hemorrhage	0	1	0	0	0
Diarrhea	0	0	1	0	0
Head discomfort	0	0	0	0	1
Hypertensive crisis	0	0	0	1	0
Upper respiratory tract infection	0	0	1	0	0

^*a*^ AE, adverse event; ESRD, end-stage renal disease.

One patient in the severe impairment group had two AEs (severe atrial fibrillation [AF] and moderate hypertensive crisis) on Day 5 post-infusion. The severe AF was categorized as a serious AE as hospitalization was required. The patient’s active medical history included the alpha-2A adrenoceptor agonist antihypertensive agent (clonidine-CATAPRESS) for hypertension, and the investigator considered it likely that the AF was precipitated by hypertensive urgency brought on by inadvertent non-compliance with clonidine-CATAPRESS on the day prior to the event. The other AEs each occurred in a single patient and were either mild (conjunctival hemorrhage, diarrhea, and head discomfort) or moderate (upper respiratory tract infection).

There were no injection-site reactions or infusion-related reactions, no withdrawals due to AEs, and no deaths reported during the study period. While a number of abnormalities in hematology, biochemistry, and urinalysis laboratory parameters and in vital signs were observed at baseline and after administration, they were considered typical for a population of subjects with renal dysfunction, and none were judged to meet the definition of an AE. There were no clinically significant changes in electrocardiograms (ECG).

## DISCUSSION

In this multi-center, non-randomized, open-label, two-part, Phase 1 study, the impact of renal impairment on the PK of nacubactam was evaluated. We found that there was a strong linear relationship between renal function and both nacubactam CL and CLr, with similar relationships observed using different measurement methodologies. Over the 24 h collection interval, total nacubactam exposures were markedly higher in subjects with impaired renal function than in subjects with normal renal function.

In the regression analyses, we found similar relationships between nacubactam clearance and renal function regardless of the method used to measure or estimate renal function. All regression models were a good fit to the data with *r*^2^ values ranging from 85% to 98%. In each case, the regression slope parameters were of a similar magnitude among the models evaluated (ranging from 0.709 to 1.372). There was no consistent pattern in the statistical significance of the covariates. When age and body weight were retained in the model, the estimates indicated a modest negative effect of age and a modest positive effect of body weight on clearance. In the two cases where sex was retained as a significant covariate, the magnitude and direction of effect was not consistent.

We also observed that nacubactam is readily cleared by hemodialysis, with more than 60% of a single dose being eliminated during a typical 4 h dialysis session. The consistency between the two methods used to estimate dialysis clearance (based on either nacubactam recovery in dialysate fluid or the extraction ratio across the dialysis machine), and the limited inter-individual variability for both (CV%: 21% and 11%, respectively), provides reassurance about the accuracy and precision of the data despite each method having several potential sources of error. For example, the collection of large volumes of dialysate for measurement of drug recovery is challenging from a practical perspective; blood flow into the dialyzer was not measured directly but was based on historical data from medical notes; and in the calculation of dialysis clearance based on the extraction ratio, the venous (outflow) concentrations were not corrected for dialysis-related hemoconcentration because the necessary measurement of total protein at the start and end of hemodialysis was not made. Conversely, as nacubactam does not appreciably distribute into erythrocytes (F Hoffmann-La Roche, data on file), there is no need to account for drug distribution into blood erythrocytes when calculating dialysis plasma clearance, which removes one source of potential error. Additionally, as the unbound fraction of nacubactum is approximately 1 (in-house data on file), the total and unbound concentrations are similar; therefore, the total concentrations instead of unbound concentrations were used in the calculations. While the sample size of three to estimate the pharmacokinetic parameters of nacubactam in healthy adults is small, because the PK parameters in normal healthy subjects have been previously estimated robustly, this sample size was, therefore, considered appropriate. The mean value in the mild group is driven by two individuals who have calculated recovery >100%. This indicates that the apparent higher cumulative recovery in the mild group is an artifact and does not truly reflect an increased urinary excretion in patients with mild renal function.

In terms of safety, a single 1 g dose of nacubactam was well tolerated in all subjects, and renal impairment had no apparent effect on the safety profile of nacubactam. The main study limitation was the use of a single dose of nacubactam in uninfected subjects. In clinical practice, treatment of infection typically involves repeated dosing over several days in medically complex patients, which could affect the AE profile. The median age of the normal group differed from the renal impairment groups (33 vs 51–73 years). Age influences renal function which is accounted for but could also influence other physiological factors; thus, some of the observed differences may be due to factors other than renal function. Another limitation of the study was that the total clearance of nacubactam in the severe renal impairment group was decreased to an extent that, ideally, the collection period should have been greater than the 24 h period. Despite this, the average AUC over 24 h constitutes up to ×83% of the AUC_0–inf_ and was sufficient to characterize the PK of nacubactam in this sub-group.

In summary, these nacubactam PK data can be used to inform dosing recommendations for patients with impaired renal function, including those receiving renal replacement therapy. There is an urgent need for new antibiotics and novel combinations to overcome current therapeutic challenges related to AMR in Gram-negative bacteria (12), and nacubactam has demonstrated promising results in the recent Phase 3 Integral-1 study (10). The finding that nacubactam is extensively cleared by renal excretion and readily removed by hemodialysis is consistent with corresponding data for other BLIs such as avibactam (13), vaborbactam (14), tazobactam (15), and sulbactam-durlobactam (16). Furthermore, the profile is similar to those of the approved β-lactam antibiotics meropenem (17), aztreonam (18), and cefepime (19), which have all been proposed as partners for nacubactam, thereby offering the potential to align the dosing recommendations for β-lactam/nacubactam combinations.

## MATERIALS AND METHODS

### Study design

This was a multi-center, non-randomized, open-label, two-part, Phase 1 study (Fig. S1). It was conducted at five investigational sites in the United States between 5 December 2016 and 22 June 2017. Subjects were assigned to a renal function group according to either estimated CL_Cr_ derived from serum creatinine concentrations at screening using the Cockcroft-Gault (CG) method (Part 1) or clinical diagnosis (Part 2). All subjects in both parts of the study received a single 1 g dose of nacubactam as a 0.5 h IV infusion on Day 1. Study drug administration occurred under medical supervision in the study clinic. No dosage modification was permitted. Subjects were discharged 24 h post-dose and returned for a follow-up visit 7–10 days later. Safety assessments were conducted on Day 1, Day 2, and at the follow-up visit 7–10 days later.

Part 1 was a parallel group design conducted in adults with stable mild, moderate, or severe renal impairment and a control group of subjects with normal renal function. The control group was matched to the renal impairment groups so that demographic characteristics (i.e., sex distribution, age, and weight) were within broadly similar ranges. Individual matching was not performed.

Part 2 of the study included a single group of adults with stable ESRD undergoing hemodialysis. Study drug administration on Day 1 started at least 90 min before commencement of hemodialysis. Hemodialysis followed the routine protocol and duration for each individual/study center.

### Population

Eligible subjects were adults aged 18–80 years at screening, weighing at least 45 kg, and with a body mass index of 18–38 kg/m^2^. All subjects provided written informed consent for study participation.

In Part 1, renal function was defined using estimated CL_Cr_ at screening derived with the CG method. Normal renal function (matched control group) was ≥90 mL/min, mild renal impairment was 60–89 mL/min, moderate renal impairment was 30–59 mL/min, and severe renal impairment was ≤29 mL/min. Adults in the normal group were required to be healthy for their age, as determined by the investigator on the basis of medical history, physical examination, clinical laboratory test results, vital signs, and 12-lead ECG monitoring. Adults in the mild, moderate, or severe impairment groups were required to have stable renal function based on two determinations of serum creatinine separated by at least seven days showing a difference ≤30% of the lower value. The severe renal impairment group could include subjects with ESRD not currently undergoing renal replacement therapy.

In Part 2, subjects with ESRD requiring hemodialysis were enrolled based on a clinical diagnosis (estimated CL_Cr_ using the CG method <15 mL/min and requiring renal replacement therapy). Subjects must have been receiving hemodialysis for >3 months at the time of the screening visit.

Exclusion criteria included renal transplantation (Part 1 only); renal carcinoma, or acute renal disease caused by infection or drug toxicity; nephrotic syndrome; hemoglobin <10 g/dL (<9 g/dL for ESRD); potassium >5.5 mmol/L; clinically significant liver disease or elevated liver enzymes; uncontrolled blood pressure; any conditions associated with intravascular volume depletion; any unstable and/or clinically significant condition rendering the subject unsuitable for study participation; recent or planned major surgery; recent history of substance abuse or addiction; presence of hepatitis or human immunodeficiency virus; current or planned pregnancy or lactation; recent blood donation or loss of >500 mL.

Medications that acutely alter creatinine concentrations through mechanisms other than alteration to the glomerular filtration rate (GFR) were prohibited. The concurrent administration of all other drugs used to manage renal failure or other chronic diseases was allowed.

### Assessments

To achieve the primary study objective of characterizing the relationship between renal function and primary PK parameters of nacubactam and to measure nacubactam clearance during hemodialysis, blood, urine, and dialysate samples from before administration until 24 h later (0.5, 1, 1.5, 2, 3, 4, 6, 8,12, 24 h) were collected. Validated bioanalytical methods (LC-MS/MS assays fully compliant with FDA bioanalytical method validation guidelines, in-house data) were employed for the quantitation of nacubactam in plasma, urine, and dialysate. In Part 1, blood and urine samples were collected for the measurement of plasma and urine concentrations of nacubactam up to 24 h post-dose. In Part 2, blood, urine, and dialysate samples were collected for the measurement of plasma, urine, and dialyzer fluid concentrations of nacubactam on Day 1. PK parameters were measured using standard non-compartmental methods. Measured CL_Cr_ was based on urine and serum creatinine measurements over 24 h prior to the start of study medication. The remaining renal function measures were assessed at Day 1, except estimated GFR based on the Chronic Kidney Disease-Epidemiology collaboration (CKD-EPI) method, which was calculated based on available cystatin C results at Day 2. Hemodialysis clearance in this study was determined via two methods. Dialysis clearance estimated from recovery in dialysis fluid was calculated using CL_D(REC)_ = Ae_HD_/AUC_HD_ (where Ae_HD_ = cumulative amount excreted in dialysis fluid during hemodialysis period and AUC_HD_ = area under the plasma concentration-time curve during the dialysis period). Dialysis clearance estimated from extraction ratio across the dialyzer was calculated using CL_D(ER)_ = BFR × (1 hematocrit) × (*C*_in_ *− C*_out_)/C_in_ (where BFR = dialysis blood flow rate taken from medical notes, *C*_in_ = arterial [inflow to dialyzer] concentration of nacubactam, and *C*_out_ = venous [outflow from dialyzer] concentration of nacubactam).

The safety and tolerability of a single IV dose of nacubactam in subjects with different levels of renal function was assessed via monitoring of AEs, laboratory safety tests (biochemistry, hematology, urinalysis), recording of 12-lead ECGs, and vital signs. AEs were categorized using the Medical Dictionary for Regulatory Activities Version 20.0.

### Statistical analysis

The sample size was determined by practical considerations and was not based on statistical power calculations; the final study size was established according to continuous assessment of the accumulated data. Results were evaluated in all study subjects who had received the dose of study medication and had drug concentration data from at least one post-dose sample.

Renal function was assessed from measured CL_Cr_ derived from urine and serum creatinine concentrations, estimated CL_Cr_ derived from serum creatinine concentrations using the methods of CG (20) and Jelliffe (21), and estimated GFR based on the Modification of Diet in Renal Disease (MDRD) (22) and CKD-EPI (23) methods. To quantify the relationship between renal function and nacubactam clearance, a linear regression analysis was performed between the log transformed CL and the log transformed estimated or measured CL_Cr_. For each analysis, the relationship was described using the regression model in the log scale with the general form: Ln(CL) = *µ* + *β* × Ln(CL_Cr_) + *ε*, where *µ* is the intercept, *β* is the slope of the regression line, and *ε* represents random error. Additional explanatory variables (body weight, sex, and age) were evaluated using a stepwise approach and retained in the regression model if statistically significant (*P* < 0.01). Model fit was assessed through examination of residual plots. In a secondary analysis, ANOVA models were fitted with each of the log transformed PK parameters AUC_0–inf_, CL, and CL_r_ as dependent variables and renal impairment group as the explanatory factor. Statistical analyses were conducted using SAS software version 9.4 (SAS Institute, Inc.; Cary, NC, USA).

## Data Availability

The data sets generated during and/or analyzed during the current study are available from the corresponding author on reasonable request.

## SUPPLEMENTAL MATERIAL

Supplemental material aac.00113-26-s0001.docx Fig. S1 to S3.

ASM does not own the copyrights to Supplemental Material that may be linked to, or accessed through, an article. The authors have granted ASM a non-exclusive, world-wide license to publish the Supplemental Material files. Please contact the corresponding author directly for reuse.
