# Supplementary material for: A multi-center, open-label, two-part study to investigate the effect of renal function and hemodialysis on the pharmacokinetics of the novel β-lactamase inhibitor nacubactam
Source: Antimicrob Agents Chemother. 2026 Apr 30;70(6):e00113-26. doi: 10.1128/aac.00113-26 (PMC13231898; doi:10.1128/aac.00113-26)
Supplement: Supplemental material — Fig. S1 to S3. [file aac.00113-26-s0001.docx]

**Supplementary data**

SUPPLEMENTARY FIG 1 Study design and subject disposition.


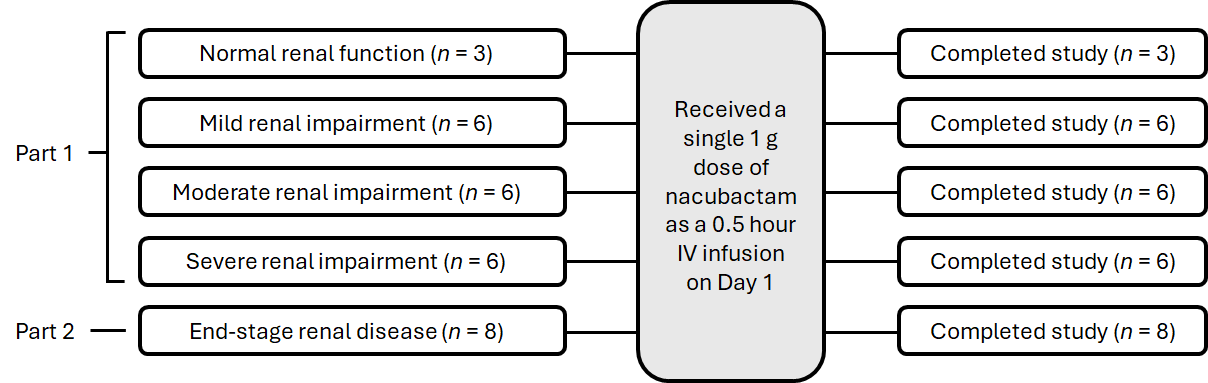


In Part 1, 21 subjects were enrolled. Normal renal function (matched control group) was defined as eCL_Cr(CG)_ ≥90 mL/min at screening, mild renal impairment as eCL_Cr(CG)_ 60-89 mL/min at screening, moderate renal impairment as eCL_Cr(CG)_ 30-59 mL/min at screening, and severe renal impairment as eCL_Cr(CG)_ ≤ 29 mL/min at screening. The severe renal impairment group could include subjects with end-stage renal disease not currently undergoing renal replacement therapy. In Part 2, eight subjects with end-stage renal disease (eCL_Cr(CG)_ <15 mL/min) and requiring hemodialysis were enrolled.

eCL_Cr(CG),_ estimated creatinine clearance using the Cockcroft-Gault method.

SUPPLEMENTARY FIG 2 Scatterplot of nacubactam V_ss_ versus mCL_Cr_ (A) and eCL_Cr(CG)_ (B) (linear scale)

A


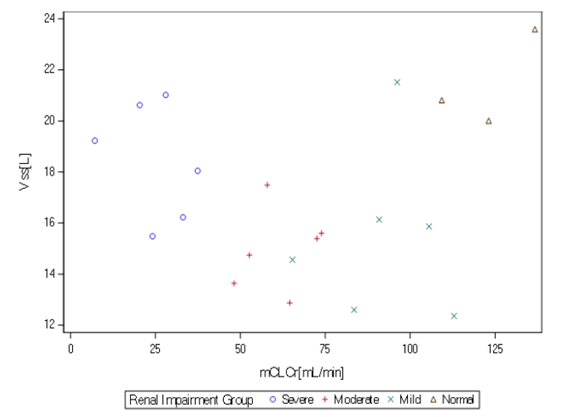


B


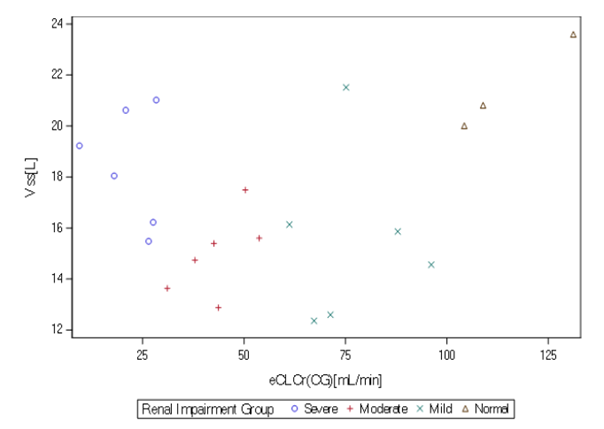


eCL_Cr_(CG), estimated creatinine clearance using the Cockcroft-Gault method; mCL_Cr_, measured creatinine clearance; V_ss_, volume of distribution at steady state.

SUPPLEMENTARY FIG 3 Estimated geometric mean ratio (GMR) for differences in nacubactam CL (A), CL_r_ (B), and AUC_0-inf_ (C) between renal function groups

A


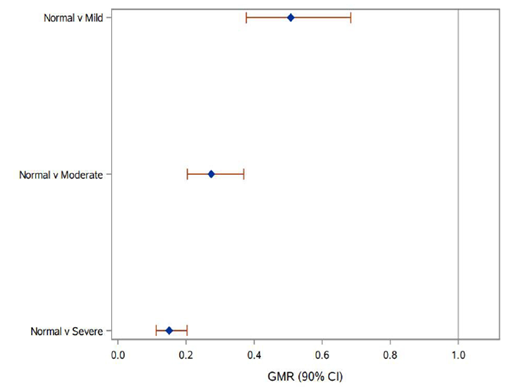


B


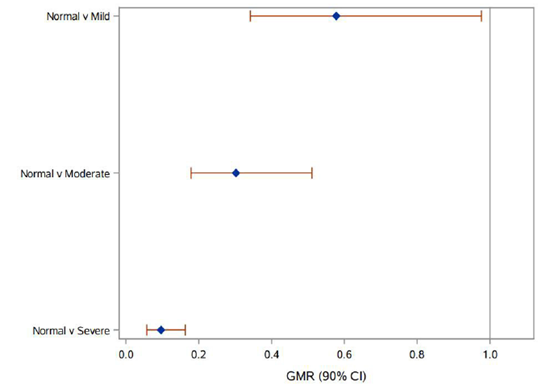


C


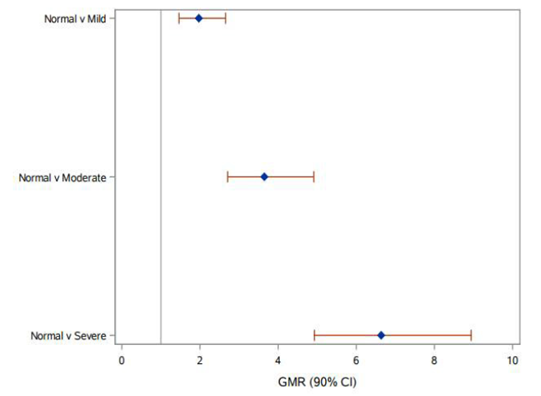


Data are shown as GMR with 95% confidence intervals.

ANOVA, analysis of variance; AUC_0-inf_, area under the concentration–time curve from time 0 to infinity; CL, total clearance; CL_r_, renal clearance; GMR, geometric mean ratio.
